# Supplementary material for: A recombinase polymerase amplification-lateral flow dipstick assay for rapid detection of the quarantine citrus pathogen in China, Phytophthora hibernalis
Source: PeerJ. 2019 Nov 18;7:e8083. doi: 10.7717/peerj.8083 (PMC6870529; doi:10.7717/peerj.8083)
Supplement: Supplemental Information 1 [file peerj-07-8083-s001.docx]

Table S1 Six pairs of candidate primers designed for the recombinase polymerase amplification-lateral flow dipstick assay

| Pair No. | Name | Sequence (5’–3’) | Length (mer) |
| --- | --- | --- | --- |
| 1 | PhRPA-F | TTCCACCCTTCCACCAGACTGCTGAGGAGG | 30 |
| 1 | PhRPA-R | TGTTAGCTGCGTGTTCGTTGGTCACCCCAGA | 31 |
| 2 | PhRPA-2F | TGTGCGTGACCCCCTTCCACCCTTCCACCAG | 30 |
| 2 | PhRPA-2R | TGTTCGTTGGTCACCCCAGAAAGGTGGAAAA | 31 |
| 3 | PhRPA-3F | TGACCCCCTTCCACCCTTCCACCAGACTGCT | 31 |
| 3 | PhRPA-3R | CAGAAAGGTGGAAAAATCTCCCTCAAACGTA | 31 |
| 4 | PhRPA-4F | CTTCCACCAGACTGCTGAGGAGGGGTCATTT | 31 |
| 4 | PhRPA-4R | AAATGTTAGCTGCGTGTTCGTTGGTCACCCC | 31 |
| 5 | PhRPA-5F | TGCTGAGGAGGGGTCATTTAATAGCAGGATA | 31 |
| 5 | PhRPA-5R | AAGTAACCTTTAAATGTTAGCTGCGTGTTCG | 31 |
| 6 | PhRPA-6F | TCATTTAATAGCAGGATAGCTGACTTATAC | 30 |
| 6 | PhRPA-6R | TGCTTGTGATCGTGCGGAAACGCTCCTGGCC | 31 |
