# Supplementary figures and images for: A recombinase polymerase amplification-lateral flow dipstick assay for rapid detection of the quarantine citrus pathogen in China, Phytophthora hibernalis
Source: PeerJ. 2019 Nov 18;7:e8083. doi: 10.7717/peerj.8083 (PMC6870529; doi:10.7717/peerj.8083)

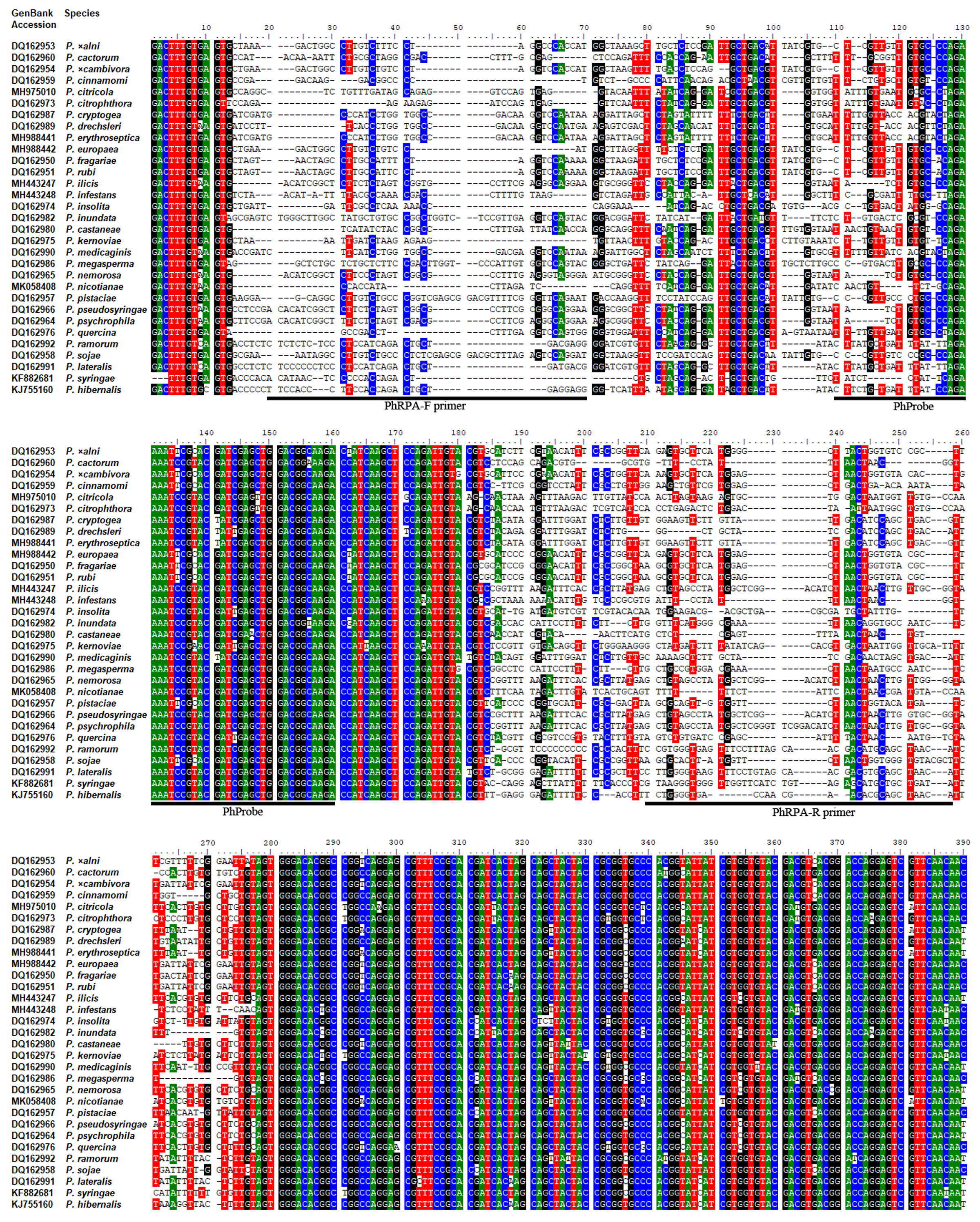

Supplement: Supplemental Information 2 — Alignment was carried out using Clustal W. Nucleotides targeted by PhRPA-F, PhRPA-R and PhProbe in the RPA-lateral flow dipstick assay are above respective lines. [file peerj-07-8083-s002.png]
